# Short-interval wildfire and drought overwhelm boreal forest resilience

**DOI:** 10.1038/s41598-019-55036-7

**Published:** 2019-12-11

**Authors:** Ellen Whitman, Marc-André Parisien, Dan K. Thompson, Mike D. Flannigan

**Affiliations:** 1 Department of Renewable Resources, University of Alberta, 751 General Services Building, Edmonton, AB T6G 2H1 Canada; 2 Northern Forestry Centre, Canadian Forest Service, Natural Resources Canada, 5320-122nd St. NW, Edmonton, AB T6H 3S5 Canada

**Keywords:** Forest ecology, Climate-change impacts, Fire ecology

## Abstract

The size and frequency of large wildfires in western North America have increased in recent years, a trend climate change is likely to exacerbate. Due to fuel limitations, recently burned forests resist burning for upwards of 30 years; however, extreme fire-conducive weather enables reburning at shorter fire-free intervals than expected. This research quantifies the outcomes of short-interval reburns in upland and wetland environments of northwestern Canadian boreal forests and identifies an interactive effect of post-fire drought. Despite adaptations to wildfire amongst boreal plants, post-fire forests at paired short- and long-interval sites were significantly different, with short-interval sites having lower stem densities of trees due to reduced conifer recruitment, a higher proportion of broadleaf trees, less residual organic material, and reduced herbaceous vegetation cover. Drought reinforced changes in proportions of tree species and decreases in tree recruitment, reinforcing non-resilient responses to short-interval reburning. Drier and warmer weather will increase the incidence of short-interval reburning and amplify the ecological changes such events cause, as wildfire activity and post-fire drought increase synergistically. These interacting disturbances will accelerate climate-driven changes in boreal forest structure and composition. Our findings identify processes of ongoing and future change in a climate-sensitive biome.

## Introduction

The circumpolar boreal zone contains approximately 30% of Earth’s forests, with one third of boreal forest land located in North America. North American boreal forests are globally important as they provide essential ecosystem goods and services, act as carbon sinks and sources, and contain extensive areas of unmanaged land^1,2^. Boreal forests typify the fire-adapted biome, having co-evolved with wildfire since the most recent retreat of glaciers. Large, infrequent, and high-intensity wildfires are the dominant disturbance in this region. Such fires are generally stand replacing, as they tend to cause widespread mortality of overstory trees and understory plants^1,3^. Climate change-induced alterations of fire regimes and the composition of post-fire boreal forests will have broad-reaching impacts on the global carbon balance and local ecologies and economies.

Observed fire frequencies, or fire-free intervals, in the western North American boreal forest range from hundreds of years to approximately 30 years between stand-replacing wildfires^4,5^. Recently burned areas in the boreal forest generally resist reburning for upwards of 30 years after a fire^6,7^, due to the lack of necessary fuel allowing fire ignition and spread^8,9^. Despite such limitations to fire occurrence, extreme fire weather conditions can override these controls, allowing fires to spread in fuel-limited recently burned areas^6,7^.

Coniferous trees dominate western Canadian boreal forests, with broadleaf tree species making up a significant but often secondary component of many stands. Adaptations to wildfire, such as vegetative regeneration, seed banking in soils, and serotinous or semi-serotinous cones are common amongst boreal plant species and allow plants and trees to rapidly repopulate burned areas following lethal fires^3,10^. These adaptations promote the persistence of species that were present prior to the disturbance, allowing stand self-replacement or “direct regeneration”, and conferring substantial forest resilience to disturbance^11^.

Although boreal forest plant species are adapted to severe wildfires, changes to fire regimes (regional characteristics of fire behaviour, ignitions, seasonality, extent, and effects) may exceed the capacity of species to regenerate following fire when pushed beyond the limits of their adaptation, compromising forest resilience^12^. Where disturbances occur in rapid succession relative to historical frequencies, boreal forest recovery may be severely limited by the lack of seed sources or bud banks^13,14^, and state-changes may occur, subverting stand self-replacement expectations and shifting the balance of vegetation types on the landscape^15^. Research suggests that the extent and frequency of wildfire, and biomass burning in western North American forests have increased under modern anthropogenic climate change^16–18^, and will continue to do so, as the climate warms^19–21^. Increasing fire activity and extreme fire weather are likely to increase the extent and frequency of short-interval reburning events, as fuel-driven controls on landscape resistance to fire are overwhelmed^6,7^.

Complicating post-fire forest recovery, climatic conditions may be increasingly inhospitable for those viable seeds that are able to establish, due to increasing drought stress^22–24^. Compounding the effects of drought stress, northern provenances of some boreal tree species are likely to be maladapted to altered climates, and demonstrate low resilience and adaptation to drought^25,26^. Although the impacts of drought on post-fire recruitment in more south-western forests have been documented^23,24^, effects of drought on post-fire recruitment in North American boreal forests are largely unexplored.

The occurrence of short-interval reburning and drought are inherently linked^7^, and these disturbances are likely to accelerate concurrently in the future, making it important to gain an understanding of how and whether fire frequency and post-fire drought in the boreal forest may interact to alter post-fire tree recruitment. The northwestern boreal forest is a hydrologically diverse landscape, with a substantial wetland component (up to 50% of land area, in some regions)^27^. It has been suggested that boreal wetlands may be resilient to fire, as forested peatlands are more resistant to shifts in tree species composition following fire than neighbouring uplands^28,29^. Hydrological feedbacks that retain elevated water tables in wetland environments, and connections between wetlands and uplands may buffer drought and climate change effects in the boreal forest^30,31^, but limited research on boreal post-fire recruitment exists that examines both productive uplands and wetland environments, where moisture stress may be less limiting.

**Figure 1:**
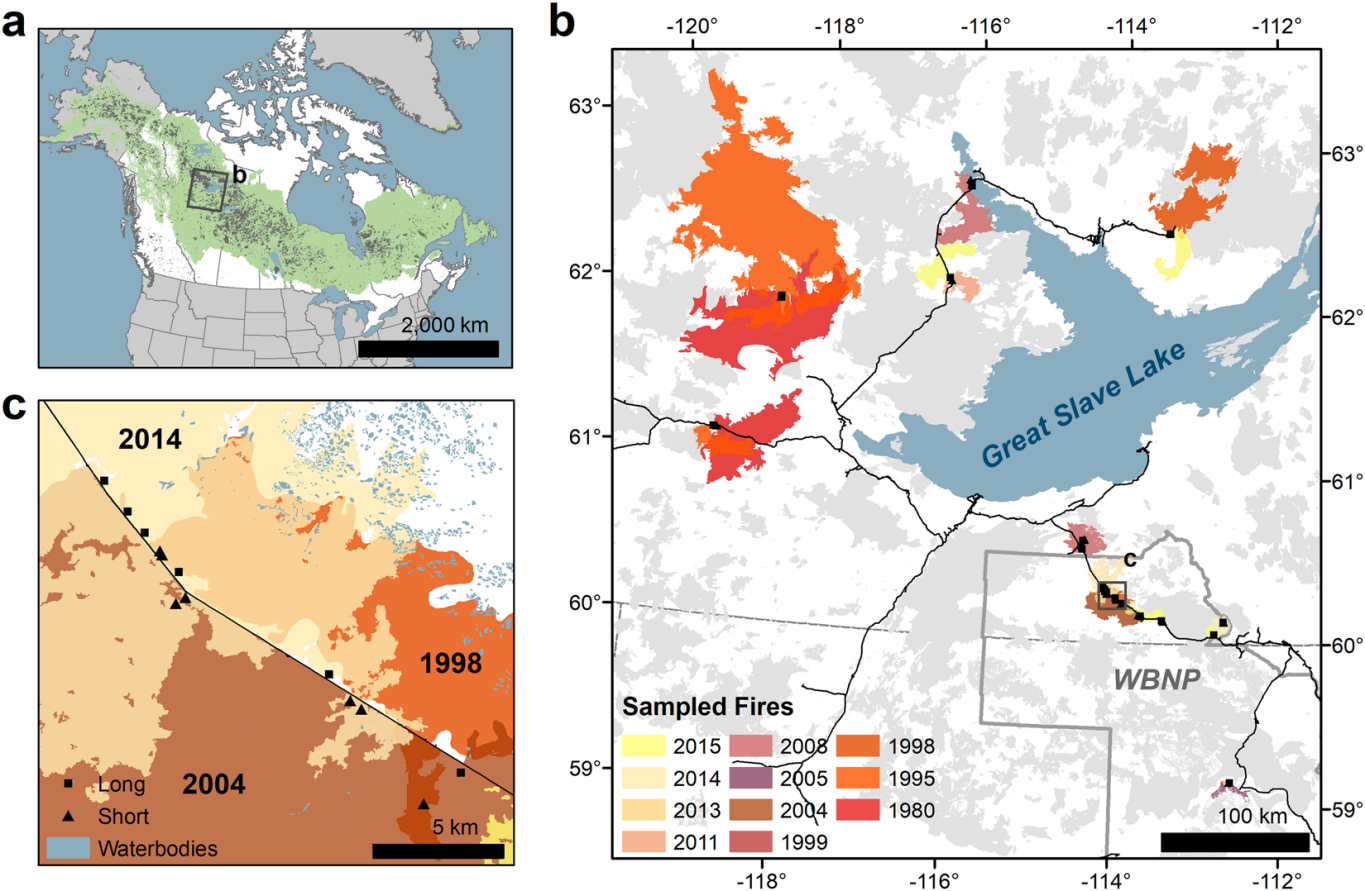
Location and fire history of the study area. (**a**) Recent (1984–2016) Canadian^67^ and Alaskan (USA)^76^ fire perimeters (grey) in the context of the North American boreal forest (green)^77^. The extent of panel (b) is indicated with a black rectangle. (**b**) The distribution of sampling sites in the study area. Sites are identified as black squares. Sampled wildfires are coloured by year of occurrence and superimposed on wildfires that occurred in the recent past (1980–2015; grey) on the landscape at the time of sampling. Wood Buffalo National Park (WBNP) is outlined in grey, major roads are shown in black, and the extent of panel (c) is indicated with a black rectangle. (**c**) Detailed area showing an example of the sampling design of paired sites with short and long fire-free intervals.

Our goal was to describe and quantify differences in forest structure and composition across a biophysical and hydrological gradient of paired sites that burned with long and short fire-free intervals (FFIs), in a range of post-fire climate conditions. We classified sites as having experienced short (≤17 years between fires) and long (≥30 years between fires) FFIs (confirmed using dendrochronological methods). The ≥30 year threshold for determining a “long” FFI was selected to represent the duration of fuel limitation of reburning in the study area^7–9^, assuming that fire weather in sites experiencing short FFIs likely overwhelmed this control^6,7^. We employed a paired study design where each long-FFI site was paired with a short-FFI site that burned in the same wildfire, and in the same pre-fire ecosite type^32^, but with a different stand origin. The primary difference between paired sites is thus the fire history of the two sites, rather than post-fire climatological conditions (pairwise correlations between site conditions reported in Table S2). By pairing sites of the same age and ecosite in this manner we are able observe persistent differences in forest structure and composition across hydrological gradients in a large study area (Fig. 1), and over a range of times post fire (1–21 years). Site pairs were closely matched in pre-fire tree species compositions, stand structure, and biogeographic settings (Table S2). We sampled sites in both uplands and wetlands with varying drainage to characterize the strength of effects along a hydrological gradient. This study design also enabled us to capture a variety of post-fire climatic conditions and moisture availability, as fires originated from seven different fire seasons, and therefore experienced diverse post-fire drought conditions. Additional details of site pairing and analyses are discussed in the methods and supplementary information. For these analyses, we created one group of all conifers and a second group of all broadleafs. Although both serotinous and non-serotinous conifers exist in this region, the majority of pre-fire conifer stems at all sites ($\bar{x}$ = 75%) were from serotinous species.

Specifically, we sought to determine whether the structure and composition of plant communities at paired short-FFI and long-FFI sites were significantly different, and whether these differences varied between well-drained and poorly-drained sites. We compared post-fire plant communities and tree seedling densities using *t*-tests and Wilcoxon signed-rank tests for paired data (adjusted for multiple comparisons) and Hill numbers equivalents. Subsequently, we used generalized linear models to characterize the relative influence of pre-fire forest structure, post-fire seedbeds, and post-fire moisture stress (drought) as drivers of observed densities and compositions of trees recruited after fire. Using these methods, we are able to quantify changes in forest structure and composition directly resulting from FFI across a range of fire histories, attribute such changes to ecological drivers, and characterize the effect of drought on post-fire recruitment in interaction with FFI in the northwestern boreal forest.

## Results

**Figure 2:**
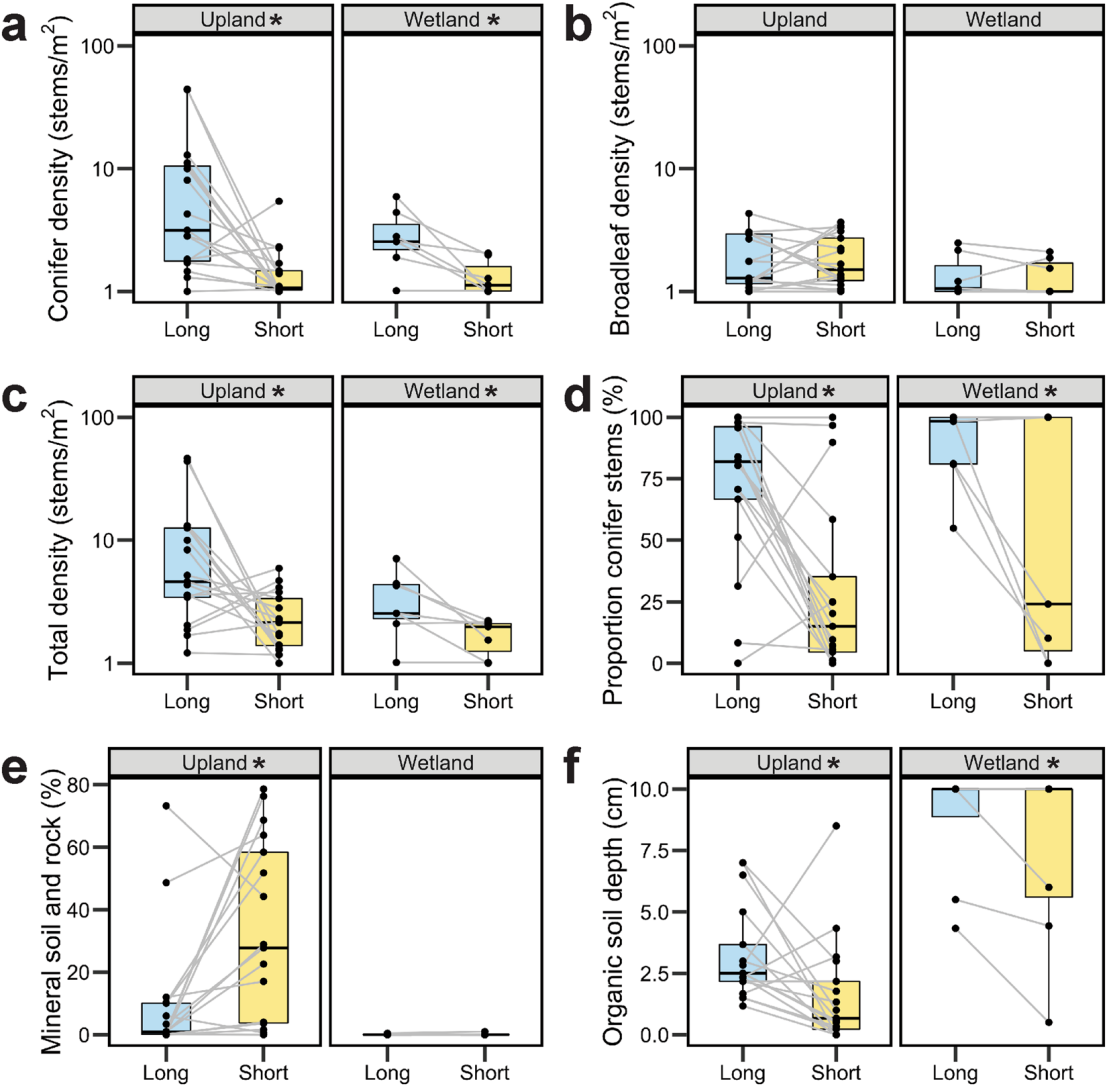
Differences between long and short fire-free interval (FFI) sites. Grey lines connect paired sites with the same elapsed time since last fire. Significance (*p* ≤ 0.05) of differences between long (“Long”; blue) and short (“Short”; yellow) pairs is indicated with an asterisk in the panel header (details of tests in Tables S3, S4, S7). Post-fire variables are: (**a**) the logarithm of conifer seedling density in the post-fire cohort, (**b**) the logarithm of broadleaf seedling and propagule density in the post-fire cohort, (**c**) the logarithm of total stem density of seedlings and propagules in the post-fire cohort, (**d**) the proportion of conifer stems in the post-fire cohort, (**e**) the mean percent cover of exposed mineral soil and rock in understory vegetation abundance plots, and (**f**) the mean depth of the residual organic soil layer.

Short-interval sites demonstrated more severe effects of fire than corresponding long-interval sites, indicated by a lower post-fire canopy density (*p* = 0.006), shallower residual organic soil (*p* = 0.010; Fig. 2), and less residual biomass of rotten coarse woody debris (*p* = 0.009) across all sites (Table S3). Upland short-FFI sites had more exposed mineral soil (*p* = 0.009; Fig. 2), and less surface cover of organic matter (*p* = 0.009), but these effects were not detected in wetlands (Table S3). Post-fire soil chemistry was also significantly different between short- and long-FFI pairs, with short-interval sites having lower mineral soil nitrogen (*p* = 0.034) across all sites.

The density and species composition of the post-fire tree cohort was also significantly different between sites with long and short FFIs. Total post-fire stem density of seedlings and propagules was significantly lower for all tree species combined (*p* = 0.007), and for conifer seedlings, specifically (*p* = 0.006; Fig. 2; Table S4). Of those seedlings that successfully established, the proportion of conifer stems in the post-fire cohort was lower following short FFIs than in long FFIs (*p* = 0.006; Table S4). On average, short-FFI pair-member sites had 24.2% lower total stem density of tree seedlings than the long-FFI member, and conifer seedling stem density was only 51.9% of that of the long-pair member. This represents an average reduction in post-fire tree stem density of 6.18 stems/m^2^.This reduction is entirely attributed to conifers; short-interval sites had an average conifer seedling deficit of 6.18 stems/m^2^ (61818 stems ha^−1^), relative to the long-interval pair-member. 72% of short-FFI sites had fewer than 50% conifer stems in the post-fire seedling and propagule cohort, whereas only 12% of long-FFI sites were not conifer-dominated. Broadleaf seedling and propagule density was not significantly different between short- and long-FFI pairs (Fig. 2; Table S4).

**Figure 3:**
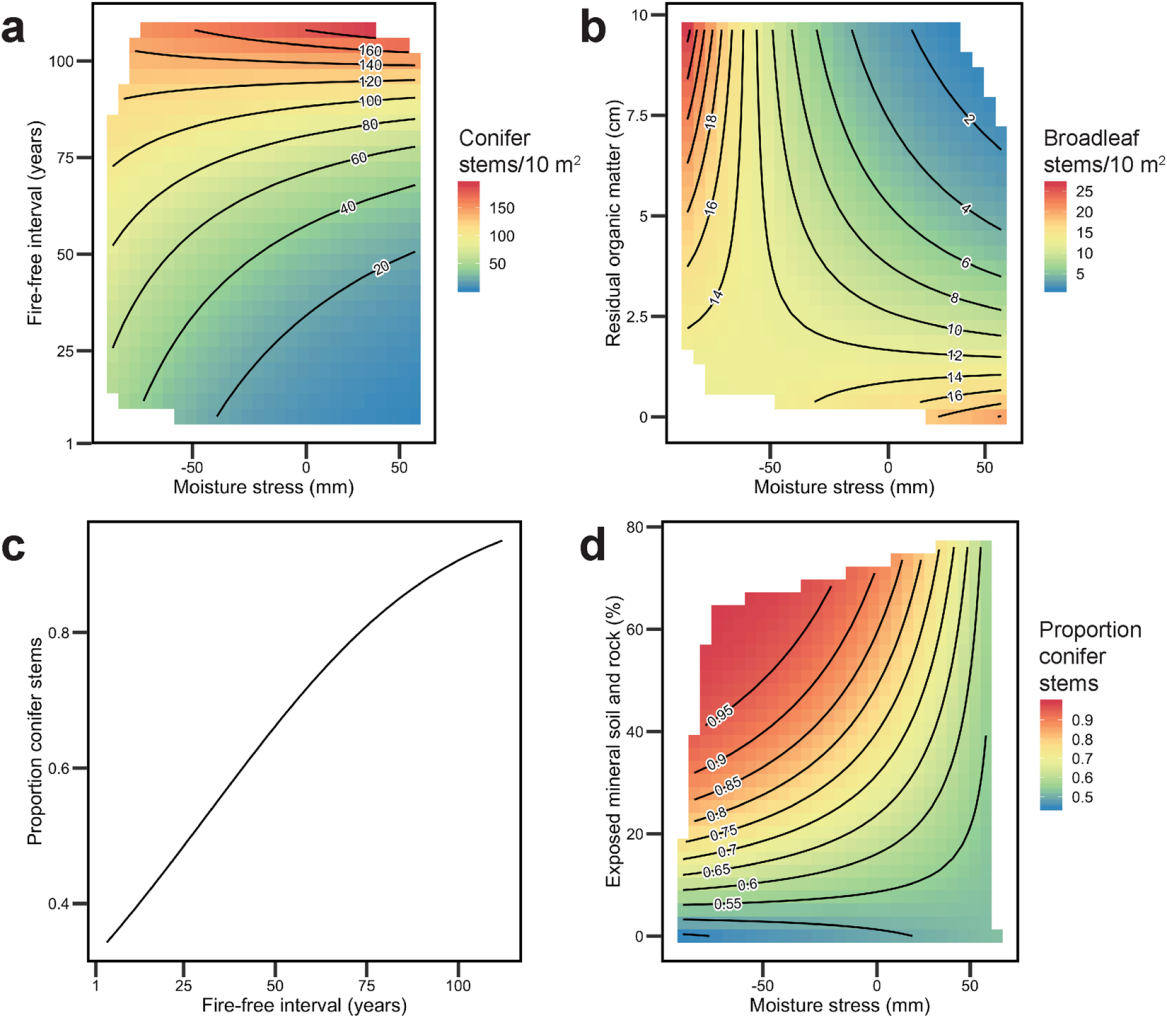
Marginal effects of fire-free interval (FFI) and moisture stress (MS) on post-fire stem density and composition of post-fire forests, in models reported in Table 1. Although models were fitted with standardized predictor variables, plot axes are labelled with observed values and units. (**a**) Effect of FFI and MS on conifer seedling density. (**b**) Effect of residual organic matter depth (RO) and MS on broadleaf stem density. (**c**) Effect of FFI on the proportion of conifer stems in the post-fire cohort. (**d**) Effect of percent exposed mineral soil and rock (%MIN) and MS on the proportion of conifer stems in the post-fire cohort.

Density and composition of post-fire tree seedling and propagule cohorts were explained by FFI and moisture stress (drought); site hydrology, time since last fire (stand self-thinning, competition, and delayed regeneration), and soil surface substrates (Table 1). We represented moisture stress using site-level cumulative anomalies of summer climatic moisture deficit in the year of fire, until four years afterward. FFI was the dominant driver of total seedling and propagule density (83% contribution to model fit), conifer seedling density (63% contribution), and the proportion of conifer seedlings (60% contribution), whereas seedbeds (exposed mineral soil (%MIN; 36% contribution) and residual organic matter depth (RO; 29% contribution)) were the most important explanatory variables in the model of post-fire broadleaf seedling density (Fig. 3; Table 1; Table S5). Time since last fire (TSF) was significant only in models of post-fire tree recruit stem density when stems were separated into broadleaf (13% contribution) and conifer groups (5% contribution).

**Table 1:** Generalized linear models of tree seedling and propagule density (stems/10 m^2^), and plot-level tree species composition.

Generalized Linear Model	Distribution	*df*	pR^2^ (CV)	RMSE (CV)	MAE (CV)	*P*
Total Seedling & Propagule Stems = 3.26 + (**FFI**^†^ × 0.74) − (**MOIST**^‡^ × 0.45) + 0.38(**FFI** × **MS**^§^) + (MS × 0.03)	Negative Binomial	44	0.59 (0.44)	56.92 (58.19)	32.02 (39.13)	<0.001
Conifer Seedlings = 2.45 + (**FFI** × 0.99) − (TSF** × 0.8) + (**%CON**^††^ × 0.73) − (**MS** × 0.68) + 0.58(**TSF** × **RO**^‡‡^) + 0.38(**FFI** × **MS**) − (RO × 0.2)	Negative Binomial	41	0.61 (0.43)	54.29 (61.69)	29.21 (39.63)	<0.001
Broadleaf Seedlings & Propagules = 0.98−2.32(**%MIN**^§§^ × BA***) − (**%MIN** × 2.13) − (**BA** × 1.4) − (**TSF** × 0.78) − (**RO **× 0.69) − 0.6(**RO** × **MS**) − (**MS** × 0.48)	Poisson	41	0.45 (0.48)	6.52 (7.26)	4.64 (5.65)	<0.001
Proportion Conifer Stems = 0.60 + (**FFI** × 1.18) + (**%MIN** × 1.02) + (**RO** × 0.98) − 0.96(**%MIN** × **MS**) − (MS × 0.4)	Binomial	43	0.49 (0.52)	0.28 (0.3)	0.22 (0.25)	0.004

Significant (*p* ≤ 0.05) predictor variables are bolded in equations. Model fits are described using averages of 10-fold cross-validated (CV) root-mean-square-error (RMSE), mean absolute error (MAE), and pseudo R^2^ (pR^2^), derived from 100 repeats. Model degrees of freedom (*n* = 49) are reported in the column *df*. Model *p*-values were derived from χ^2^ tests of model deviance explained relative to a null model.

^†^FFI: Fire-free interval (years).

^‡^MOIST: Site moisture (from subxeric to subhydric).

^§^MS: Moisture stress (mm), as represented by site-specific cumulative anomalies of summer climatic moisture deficit (CMD) over 0 – 4 years post-fire.

**TSF: Time since last fire (years) at time of sampling.

^††^%CON: Proportion of conifer trees in the pre-fire cohort (%) calculated from basal area (m^2^ ha^−1^).

^‡‡^RO: Residual organic matter depth (cm).

^§§^%MIN: Proportion of exposed mineral soil and rock (%) at the surface.

***BA: Pre-fire basal area of trees (m^2^ ha^−1^).

Post-fire moisture stress (MS; climatic moisture deficit anomalies) significantly influenced stem density of the post-fire tree seedling cohort, but was a substantially less influential driver of post-fire recruitment than FFI (0.2–13% contribution; Table S5). Often MS was only significant in models as an interaction with FFI or seedbed variables; however, such interactions were significant, improved model R^2^ and reduced error, and, when considered separately from the contribution of main effects, were important (Fig. 3; Table S6). Conifer and total stem density increased with increasing FFI, and this effect interacted with MS. Where FFIs were short and post-fire MS was high, seedling density was low. This negative effect of MS declined with increasing FFI length (Table 4.1, Fig. 3). Overall, moisture stress negatively affected post-fire broadleaf stem density; however, MS interacted with RO depth. On thin residual organic layers MS led to increases in broadleaf seedling density (Fig. 3). The proportion of conifer seedlings in the post-fire cohort increased with increasing FFI (Fig. 3). Increasing availability of mineral soil substrates (%MIN) also increased the proportion of conifer seedlings in the post-fire cohort, but this effect was dampened where moisture stress was high, favouring instead increases in the proportion of broadleaf species (Fig. 3).

Models of seedling density and composition provided some evidence for different post-fire outcomes in uplands and wetlands. Although thin (i.e., post-fire) residual organic layers were initially positive for conifer seedling recruitment, stand self-thinning occurred over time in upland environments ($\bar{x}$ RO = 2.4 cm), whereas conifer seedlings continued to establish in wetlands ($\bar{x}$ RO = 8.1 cm) over time (Fig. S1). Although years with low moisture stress increased broadleaf establishment in sites with deep organic layers (wetlands), higher moisture stress reduced broadleaf stem density in such environments (Fig. 3). The proportion of conifer stems generally increased with increasing RO depth (Fig. S1).

We also observed meaningful differences between short- and long-FFI sites in the post-fire communities of understory vascular plants and shrubs. Short-FFI sites had less vegetation cover of herbaceous understory plants (*p* = 0.034), and of forbs specifically (*p* = 0.021; Table S7). We identified small evergreen shrubs, shallow resprouting species, and seed-banking species as indicator species in long-FFI uplands using multilevel pattern analysis^33^. The only significant indicator species identified in short-FFI uplands was downy wildrye (*Leymus innovatus* (Beal) Pilg.), a grass with deep rhizomes (Table S8). Indicator species in long-FFI wetlands were evergreen shrubs and species that resprout from shallower rhizomes, whereas indicator species in short-FFI wetlands were generally fire-tolerant resprouting species^34^ (Table S8).

**Figure 4:**
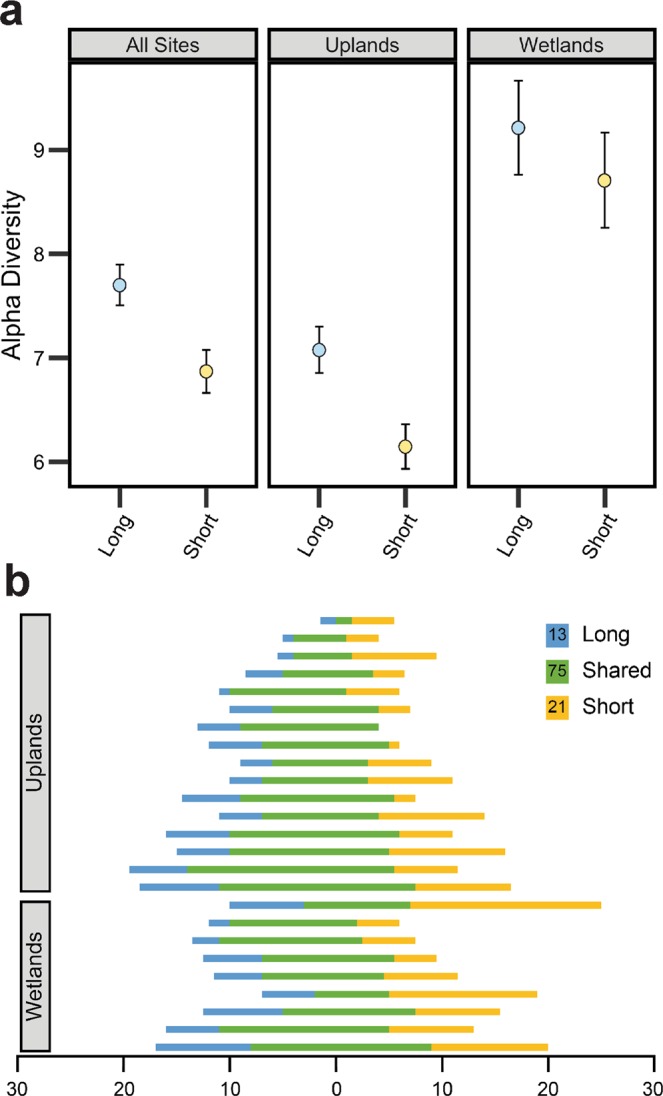
Differences in understory plant community diversity observed at sites with long and short fire-free intervals (FFI). (**a**) Differences in effective alpha diversity (Hill numbers equivalents, Shannon measures) of understory plant communities and bootstrapped 95% confidence intervals for the mean between long-FFI (“Long”; blue), short-FFI (“Short”; yellow) sites, derived from 999 iterations. (**b**) Richness of shared and unique vascular plant species between paired long (blue) and short (yellow) FFI sites. Species shared by paired plots are represented in green. The total of unique and shared species across all sites is reported in the legend.

Alpha diversity (bootstrapped Hill number equivalents, Shannon measures^35^) was significantly higher by approximately one effective species in long intervals than in short intervals across all sites, and especially within uplands (Fig. 4). This is equivalent to an 11% decline in diversity (13% in uplands). Effective beta diversity was higher in short-FFI wetlands than long-FFI wetlands, and homogeneity was lower in short-FFI wetlands. The same trends were present across all sites and in uplands, but differences were not significant (overlapping 95% CI; Fig. S2). Although all pairs of sites shared common species, we often observed unique species in either pair member (Fig. 4). Some species were unique to either long- or short-FFI sites; however, these were rare individuals that only occurred in one or two sites.

## Discussion

Short-interval reburns in northwestern Canadian boreal forests lead to different understory plant communities, tree species compositions, and stem densities of trees recruited post-fire than those observed at paired sites that burned in the same recent fire but with a longer FFI. Paired sites experienced the same post-fire climatological conditions (Table S2) and had the same length of time to recover and revegetate, yet differences between paired sites were persistent and significant, indicating that they are attributable to the different fire histories of the two groups. Although FFI was the primary driver of post-fire forest composition and structure, the occurrence of drought in the years immediately post-fire further reduced forest resilience to short FFIs.

### Differences in post-fire ecology as a result of fire history

The structure and composition of post-fire tree cohorts are significantly different between short- and long-FFI sites. Short FFIs led to more open forests with a reduced conifer component due to a reduction in conifer recruitment, similar to trends observed in other western boreal forests^36^. Longer FFIs had an overall positive effect on total stem density of trees, conifer seedling density, and the proportion of conifer stems post-fire. The dominant importance of FFI to post-fire stem densities of conifer seedlings suggests that the primary mechanism for changes in conifer seedling establishment was a lack of seed sources due to tree and sapling mortality and combustion of immature or non-serotinous cones. The pre-fire proportion of conifer stems and pre-fire basal area (proxies for seed sources for both obligate live seeders and serotinous conifers), and seedbeds were less important than FFI. This variable likely captures interactive effects of both reduced seed availability and lesser seed viability due to immaturity^13,36^. For the purpose of this study, we aggregated serotinous and non-serotinous conifers. These two groups are likely to respond differently to disturbances, as non-serotinous boreal conifer species require live seed sources. These different responses to fire may be apparent in the increasing stem density over time after fire observed in wetlands, where black spruce (*Picea mariana* (Mill.) Britton, Sterns & Poggenb; a semi-serotinous conifer), as well as white spruce (*P. glauca* (Moench) Voss) and tamarack (*Larix laricina* (Du Roi) K. Koch; both obligate live seeders) were more dominant. Despite these differences, the lack of all conifer seed sources, either from live trees or released from serotinous aerial seedbanks, at short-FFI sites would affect recruitment negatively, with a possibility for better regeneration at reburn edges for both life-history groups^37^.

Short-FFI sites had substantially thinner residual organic layers and increased exposed mineral soil. Mineral soils and thin organic layers within fires are found to promote conifer regeneration^38,39^, and yet, despite the higher availability of such seedbeds in short-interval sites, overall stem density of conifers was lower following short FFIs. Broadleaf seedling density was more related to seedbeds than FFI, with increasing broadleaf establishment on thinner residual soils^40^. The light weight of broadleaf seeds allows for extensive dispersal capabilities and these species are not meaningfully limited by seed availability from nearby seed sources^41,42^. As FFI directly affects soil substrate drivers (Table S3), this alternate pathway reinforces the importance of FFI as the primary determinant of post-fire recruitment. Patterns of density and composition of post-fire forests are established shortly after a disturbance in the boreal forest^43^, suggesting that a reduction in the dominance of conifers and more open forest structure developed following repeated fires will persist into the future.

Research has suggested that broadleaf trees (particularly aspen (*Populus tremuloides* Michx.)) may replace conifers where they are threatened by climate change and shortened disturbance intervals, in more southerly forests^42,44^. The proportion of stems contributed by broadleafs at the sampled sites was higher following short interval reburns, offering support for this conclusion. However, despite their increase in dominance due to the decrease in conifer establishment, broadleafs did not increase in abundance in short-interval sites, resulting in a net decline in total stem density. Stem thinning of broadleaves continued as time since fire elapsed (Fig. S1), leaving both forest structure and composition fundamentally altered by shortened FFIs in northwestern boreal forests^36,43^. Projections of future climates in western boreal uplands suggest arid climates and frequent fire will limit the expansion of broadleaf forests, favouring instead the expansion of grasslands^45^. Furthermore, broadleaf-dominated forests resulting from short FFIs in northwestern Canada may be susceptible to extensive drought-induced mortality of aspen already observed in nearby dry southwestern boreal forests^46^, raising questions about how persistent aspen-dominated short-FFI forests may be.

Despite adaptations to lethal wildfire amongst boreal plants, the understory vascular plant communities that established following short FFIs were effectively less species rich and had a lower abundance of herbaceous vegetation. Differences in understory plants appear to be largely the result of soil heating and combustion, as indicator species of long interval sites were fire-intolerant or tolerant only of lower-intensity fire that allowed seedbanks and vegetative propagules to persist. In uplands, indicator species at long-FFI sites were forest species, whereas the only significant indicator of short-FFI uplands was a grass. Altered germination substrates and soils (exposed mineral soil, reduced organic matter, and soil nitrogen) at short-FFI sites may also have proven inhospitable to herbaceous species establishing from seed, favouring resprouters and nitrogen-fixing species instead. Such changes represent a fundamental shift in the forest understory, with an increased presence of deciduous and resprouting understory species, but an overall decline of herbaceous vegetation cover^47^.

Variability in burn severity of both the most recent and prior fire is likely important to the strength and influence of fire interval effects on post-fire forests. Earlier fires prime forests, partially determining the availability and type of total biomass, propagule and seed sources, and fuel structure and composition^43,48,49^. The sampled short-FFI sites had greater post-fire canopy openness^47^ and thinner residual organic layers^13,50^. Short-FFI sites also had reduced coarse woody debris (nurse logs^51^), which is important for delayed regeneration of some non-serotinous boreal conifers^52^. Despite the dominance of large, high-mortality crown fires in the northwestern boreal forest, surface fires and intermittent crown fires also regularly occur, depending on fire weather and fuel structure (e.g., sandy jack pine (*Pinus banksiana* Lamb.) uplands^10,49^). The effects of successive low-severity short-interval fires would be unlikely to lead to changes of the same magnitude and effect we report^48^.

### Upland and wetland resilience

Wetlands burn less severely^49,53^ and subsequently resist post-fire shifts in tree species dominance^28,29^, indicating that they may be more resilient to disturbance from wildfire than uplands. Amongst our sites, effects of repeated fires and differences in short and long FFI post-fire tree and understory ecology were more pronounced in uplands, suggesting that changes resulting from shortened FFIs may occur more rapidly at well-drained sites. Soil and seedbed substrates in wetland sites (deep residual organic layers) also promoted conifer dominance, and establishment of conifer trees continued in such sites over time, whereas sites with thin or no residual organic layer (uplands) underwent thinning of conifer trees over time (Fig. S1). Continuing post-fire conifer establishment in wetlands may be due in part to lower severity fire providing refugia for obligate live seeder species^49^. Despite this evidence for wetland resistance to change relative to uplands, short-interval reburned wetlands experienced similar reductions in stem density and conifer seedling dominance to those observed in uplands, leading to a lowered overall conifer biomass. Such changes are consistent with a reduction in conifer seed availability and viability^13,36^.

### Potential climate change effects and interactions

The sites sampled for this research have already experienced a substantial recent increase in mean annual temperature, with an average temperature anomaly of +1.6 °C in 2017^54^; more than double the observed global average anomaly of +0.7–0.9 °C (Reference period 1981–2010^55^). In the study area, a simultaneous increase in precipitation mitigated potential increases in moisture stress in recent years (Fig. S3)^54,56^. Increases in precipitation are expected to slow in coming decades and the proportion of precipitation occurring in the summer to decrease. Increases in temperature are likely to outpace increases in precipitation, leading to an increasingly dry climate and more severe fire weather and activity^19,20,57,58^ (Fig. S3), as well as potential for significant regional drought in the western boreal forest^59^. Climatic moisture deficit (precipitation - reference evaporative demand; CMD) anomalies, relative to 30-year normal of CMD, are highly correlated with annual area burned in the study area (Fig. S4), suggesting that both the primary stand-initiating disturbance and subsequent post-fire moisture stress are likely to increase concurrently.

The occurrence of a drought reduces the strength of the spread- and ignition-limiting effect that young stands have on fire occurrence, and contributes to years with widespread fire^6–8^. Increasing drought will not only make fires more frequent and more severe, overriding stand-age limitations to fire, but will also further alter the forests that regenerate by influencing post-fire recruitment^23,24^, as demonstrated in this study. Moisture stress had an additional negative effect on both conifer and broadleaf seedling density. The influence of drought on conifer seedling density was particularly pronounced in sites that had experienced short-interval reburns (Fig. 3), indicating that drought further decreases forest resilience to short-interval reburning. Synchronous disturbance from short-interval fire and subsequent drought may substantially limit conifer seedling recruitment, accelerating forest change.

Severely burned surfaces and exposed mineral soils are thought to provide the best substrate for seedling establishment and survival of the dominant boreal tree species^39,40^. Greater abundance of mineral soil surfaces increased the proportion of conifers in the post-fire cohort in periods of low or zero moisture stress, and decreased conifer dominance in drought conditions. Severely burned surfaces reduced the density of broadleaf stems; however, post-fire drought slightly increased their abundance in such areas (Fig. 3). This shift in the effect of soil burn severity on broadleaf seedling density may be due to reduced competition from conifers, which also fared poorly under drought conditions, especially where surface burn severity was high. Although thick organic layers are generally limiting for broadleaf establishment due to fluctuations in temperature and rapid surface drying^41^, the increased moisture availability in wet years appears to have conferred an temporary advantage to broadleafs establishing on thicker soils. Drought appears to reinforce the dominant fire-interval-driven changes in forest composition and structure, leading to an increased proportion of broadleaf species in the post-fire cohort and lower stem density. Long-term monitoring at permanent plots has recorded ongoing biomass declines from increasing mortality of mature trees due to climate change and increasing moisture stress^60^. Our more limited data suggest that the post-fire establishment density of boreal trees is also likely to decline in response to both fire frequency and drought. Concurrent with ongoing biomass declines in mature forests, this reduction in the density of tree recruitment has implications for reduced biomass accumulation over time in young stands.

It is unknown whether the rate or extent of short-interval reburning is increasing in the northwestern boreal forest, but as the duration, area burned, and frequency of fires increase in the future it is likely that active wildfires will continue to breach recently burned young forests despite fuel limitations and vegetation feedbacks^6,15^. The role of severe drought in both the occurrence of short-interval reburning^6,7^, and the post-fire outcomes for juvenile trees^23,24,39^ suggests that climate change will have multiple direct effects on the structure and composition northwestern boreal forests as repeated wildfires and drought interact, although more research is needed to understand this relationship. The piecemeal occurrence of short-interval reburns in interaction with drought may serve to accelerate widespread climate-driven changes in forest openness and composition, that are projected and ongoing in much of the boreal forest^45,61,62^.

This research contributes to mounting evidence that repeated and interacting natural disturbance events can fundamentally alter the structure and composition of boreal forests, despite widespread adaptations to fire. We also provide some evidence that wetland environments may confer a moderate degree of resilience to fire, when fire frequency is increased. Interactions with climate, mediated through drought, appear to amplify the effects of shortened fire intervals that cause species composition shifts and increase forest openness, suggesting that modelled rates of compositional change and regeneration failure driven by fire frequency (e.g.^15,62^), but not drought, may be conservative. If the frequency and extent of short-interval reburning in the northwestern boreal forest increases with future climate change it is likely to accelerate and reinforce climate-driven shifts towards open forests with a reduced conifer dominance as forests’ adaptive resilience to disturbance is overwhelmed.

## Methods

### Study area

The northwestern Canadian boreal forest (Fig. 1a) is an expansive forest biome characterized by short, warm summers, and long cold winters^63^. Forests of this region are dominated by coniferous tree species, primarily black spruce (*Picea mariana*) white spruce (*P. glauca*), and jack pine (*Pinus banksiana*). Broadleaf tree species, largely trembling aspen (*Populus tremuloides*), often co-occur with conifers^63^. Large, stand-replacing wildfires are the dominant disturbance in this region^3,63^, recurring approximately every 69 (palaeoecological data^4^) to 160 years (map data^5^). The study area has a substantial wetland component, with forested peatlands covering as much as 50% of the landscape^27^.

### Data collection

In 2016 we sampled 50 sites (25 pairs) around Great Slave Lake that had burned in the last 21 years, between the years of 1995 and 2015, in the Northwest Territories and Alberta, Canada (Fig. 1; Table S1). Twenty-five sites had a short recent FFI ranging from four to 17 years ($\bar{x}$ = 10.6 years). These sites were paired with 25 sites with longer FFIs ranging from 30 to 112 years ($\bar{x}$ = 64.0 years; Table S1). Of these 25 pairs, eight were wetlands and 17 were uplands. Fire intervals in poorly drained boreal forests are typically longer than those observed in uplands, promoting the persistence of semi-serotinous black spruce (*Picea mariana*) and reducing the likelihood of short-interval reburning^64^. This likely contributed to our difficulty in finding short-interval wetland sites, which limits the certainty of our results in the wetland group.

Sites were located ≥100 m from roads. We accessed some sites by helicopter. At each site we sampled live and dead mature tree (>1.33 m height, diameter at breast height (DBH) ≥ 3 cm) stem density, basal area, and species composition using the point-centred quarter method^65^ every 5 m along a 35-m transect oriented north-south, beginning at 0 m. On the west side of the transect at the same eight points we sampled vegetation abundance of understory species and small shrubs (≤0.5 m) in 1 × 1 m quadrats, and estimated the percent cover of exposed organic and inorganic surface substrates. We sampled the density of tall shrubs using a variable-length (17.5–35-m, depending on shrub density) 2-m wide belt transect on the east side of the transect. Individual shrubs were defined as identifiable shrub genets or clones, with a height ≥0.5 m. Percent exposed mineral soil and organic matter were averaged to produce one percent cover value for each metric, per site. We sampled tree seedling and sucker (resprouting stems from top-killed individuals) stem density, species, and status (live or dead) on the east side of the transect using a 2-m wide belt transect with variable lengths depending on height classes with minimum transect distance increasing with tree height (<0.1 m – 10-m transect; <0.5 m – 20-m transect; ≤1.33 m – 35-m transect). Where stem density or size classes were highly uneven, we extended the length of the belt transect for smaller size classes, up to a maximum of 35 m. We measured sapling (trees >1.33 m in height but with a DBH < 3 cm) density, species, and status for the entire 2 × 35-m transect. At 0, 17.5 and 35 m along the transect we sampled the depth of the soil organic layer and measured overstory canopy density using a convex spherical densiometer. We averaged residual organic layer depth measurements for each site. We measured surface fuel and coarse woody debris biomass loading along the same 35-m transect and over an additional 15 m, resulting in a 50-m long transect^66^.

At each site we sampled fire-scarred trees from the pre-fire cohort to verify recent FFIs suspected from fire history maps^67^, and stand age at the time of the most recent fire. If multiple scarred trees were available, we sectioned up to four individuals per site. Live trees or trees killed in a known recent (<5-year-old) stand-replacing fires were preferred; however, if no live scarred trees were available, a section of a nearby live tree of the same species was used to cross-date dead fire-scarred individuals. We sanded tree sections using a belt sander and a fine-grit sandpaper, digitally scanned each section, and counted and measured annual rings and dated fire scars on the scanned images in CooRecorder^68^.

We used ClimateWNA to downscale PRISM climate data (available at tinyurl.com/ClimateWNA; methodology described in^54^) to site elevations derived from the Canadian Digital Elevation Model^69^. For each site, we estimated cumulative post-fire moisture stress by summing annual anomalies in summer (JJA) climatic moisture deficit (reference evaporative demand - precipitation (mm); CMD) for the year of fire and all summers afterward, up to a maximum of three years post-fire (4 years total). Where sites had burned fewer than three years in the past at the time of sampling, we calculated cumulative anomalies using only the year of fire and intervening years (2–3 years total). Anomalies were the difference between the actual summer CMD of each year, and mean summer CMD for the 1961–1990 normals period (all sampled fires occurred after 1990). To best represent the effect of moisture stress on tree recruitment we assessed all combinations of cumulative years after fire beginning either year 0 or year 1, and ending 2, 3, and 4 years post-fire; by which time approximately half of seedling establishment has likely occurred^43^. Possible moisture stress variables were correlated and generally had similar significance and effects in models; however, we chose 0 to 4 years as this window had the best improvement in some model fits and was significant in all models.

We pooled all coniferous tree seedlings for analyses. All broadleaf seedlings and suckers were also pooled, to characterize general trends in species composition. We described the pre-fire species composition using all live and dead dominant overstory trees measured in the point-centred quarters, as well as any dead saplings (>1.33 m height <3 cm DBH), with the assumption that such individuals were killed in the recent fire. This method allowed us to estimate pre-fire tree species composition of regenerating cohorts in young stands that experienced short FFIs and had no or few remaining identifiable mature trees. Although live seedlings <1.33 m in height may have originated before the fire, it is possible for broadleaf suckers and some jack pine seedlings to grow to this height within the sampling window (≤21 years post-fire); therefore, these individuals were not included in estimates of the pre-fire cohort.

### Statistical analysis

All statistical analyses were conducted in R^70^. We identified significant differences between short and long FFIs using statistical tests for paired data. If the difference in a variable between the short- and long-FFI pairs was normally distributed, we used a paired *t*-test. If the differences were non-normally distributed, we used a nonparametric paired Wilcoxon signed-rank test. Paired statistical tests were conducted for all sites pooled, and in subgroups of wetlands, and uplands. If tests for all three groups are significant, we report the significance of the total dataset. If only two tests were significant (the total dataset and a subgroup), we report only the test with the higher *p*-value. We adjusted *p*-values to account for the false discovery rate, due to our use of multiple statistical tests^71^.

To characterize drivers and controls of post-fire seedling recruitment we fitted generalized linear models (GLMs) explaining the density of tree seedlings and propagules, and post-fire proportions of conifer stems as a function of proposed drivers of post-fire moisture stress, fire history, soil substrates, pre-fire forest structure and composition, and stand age at the time of sampling. Total seedling and sucker density, conifer seedling density, and broadleaf seedling and sucker density (stems/m^2^) were multiplied by 10 and rounded to produce integer values, approximating stem counts per 10 m^2^. We assessed the form of relationship between the independent and dependent variables and found no non-linearity. Sites were assessed as individual observations in the GLMs, as opposed to pairs. Before fitting models, we standardized independent variables. To account for overdispersion in seedling and propagule count data we used negative binomial and Poisson distributions for these models, whereas the conifer proportion of the post-fire cohort was represented with a binomial distribution. Models were fitted using the caret package^72^, and model fits were assessed using pseudo R^2^ (the squared Pearson correlation between observed and predicted values from models), mean absolute error (MAE), and root mean square error (RMSE) from full and 10-fold cross-validated model statistics derived from 100 repeats. Cross validation was employed to account for the small sample size and to limit possible overfitting due to substantial outliers in seedling count data. We retained independent variables in models only if they were significant. We excluded collinear independent variables (Spearman’s ρ > 0.7), retaining the correlated variable that was more significant of the two. Significance and standard errors of independent variables was determined using White’s estimator in the sandwich package^73,74^ due to significant heteroscedasticity. Having identified a parsimonious model for each dependent variable, we iteratively removed each independent variable to examine their relative importance (% contribution to ∆AIC).

Finally, to examine the effect of fire intervals on understory vegetation, we characterized understory species diversity and abundance using bootstrapped Hill numbers equivalents (effective numbers of equally-abundant species necessary to give the same value of a diversity measure^35^) calculated in the vegetarian package with Shannon measures^75^. We identified unique indicator species (single site-groups) of short- and long-interval wetlands and uplands using multi-level pattern analysis in the indicspecies package^33^.

## Supplementary information

Supplementary Information

## Data Availability

Data is available at 10.5281/zenodo.3558302.
