# Supplementary material for: Short-interval wildfire and drought overwhelm boreal forest resilience
Source: Sci Rep. 2019 Dec 11;9:18796. doi: 10.1038/s41598-019-55036-7 (PMC6906309; doi:10.1038/s41598-019-55036-7)
Supplement: Supplementary file 1 — Supplementary Information [file 41598_2019_55036_MOESM1_ESM.docx]

# Short-interval wildfire and drought overwhelm boreal forest resilience

Ellen Whitman, Marc-André Parisien, Dan K. Thompson, Mike D. Flannigan

**Field data limitations and sampling details**

All sampled sites were paired with a site burned in the same wildfire and year, with the exception of one pair. The long FFI member of this pair burned in a holdover of the same fire, one year after the short member site burned. Generally, the more recently an area has burned, the lower the seedling density and vegetation cover, and the higher the observed soil burn severity. Because our results suggested that these characteristics are associated with short FFIs we were comfortable with this bias, as it did not exaggerate the effect of a short interval, but rather dampened the effect of a long one. We paired two short FFI sites with the same long FFI site, which we sampled in the same ecosite and wildfire as the short FFI pair-members. The understory tree data from one wetland site, and shrub data from two sites (one upland and one wetland) were lost due to a technical error with data collection equipment. We excluded both pair-members from paired analyses of differences between sites.


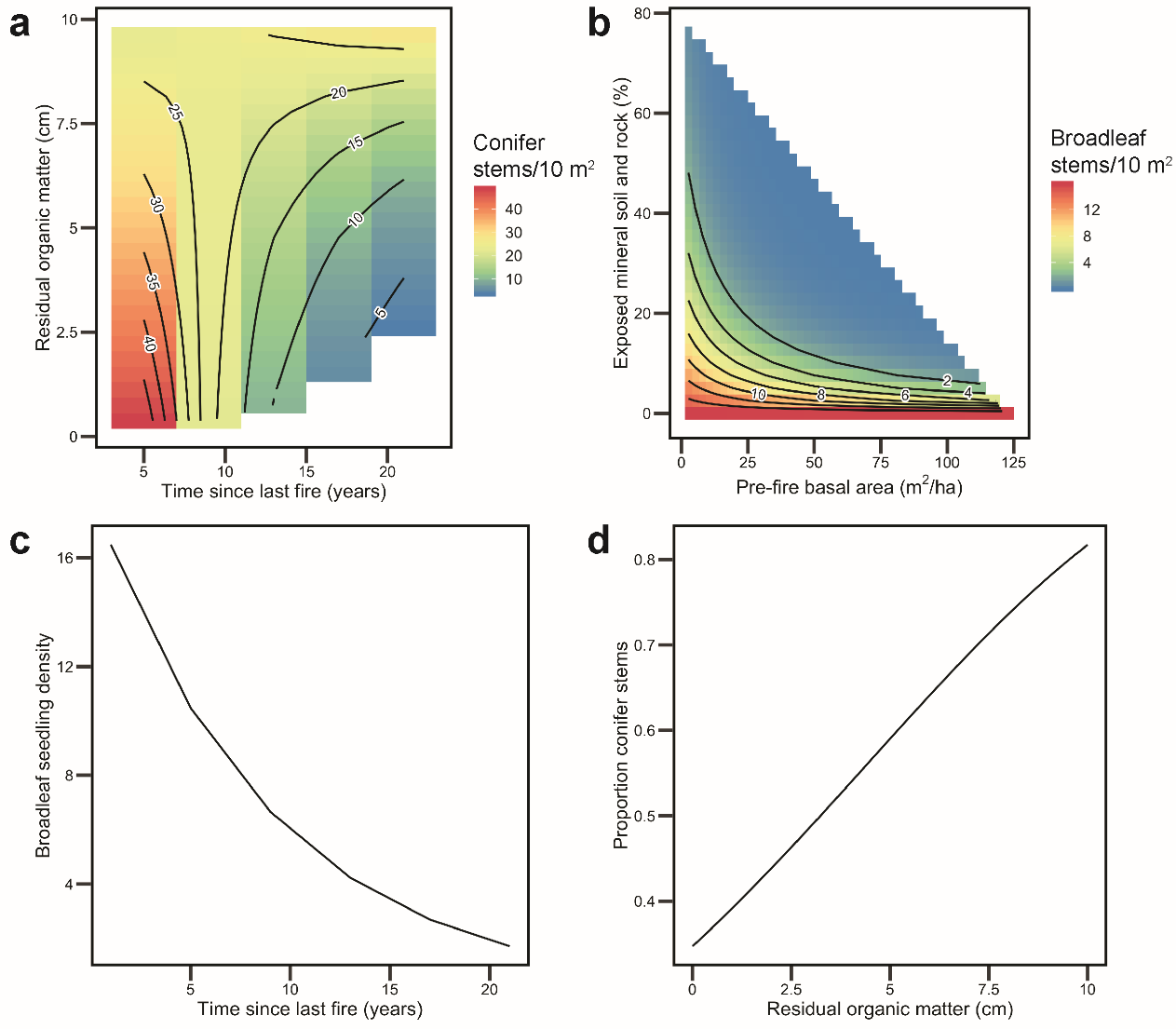


Figure S1. Marginal effects of residual organic matter depth (RO) and time since last fire (TSF) on post-fire stem density and composition of post-fire forests. Deep organic layers (≥ 8 cm) are associated with wetlands, whereas thinner organic layers were largely restricted to uplands. Although models were fitted with standardized predictor variables, plots axes are labelled with observed values and units. (a) Effect of RO and TSF on conifer seedling density. (b) Effect of RO and MS on broadleaf stem density. (c) Effect of TSF on broadleaf stem density in the post-fire cohort. (d) Effect of RO on the proportion of conifer stems in the post-fire cohort.


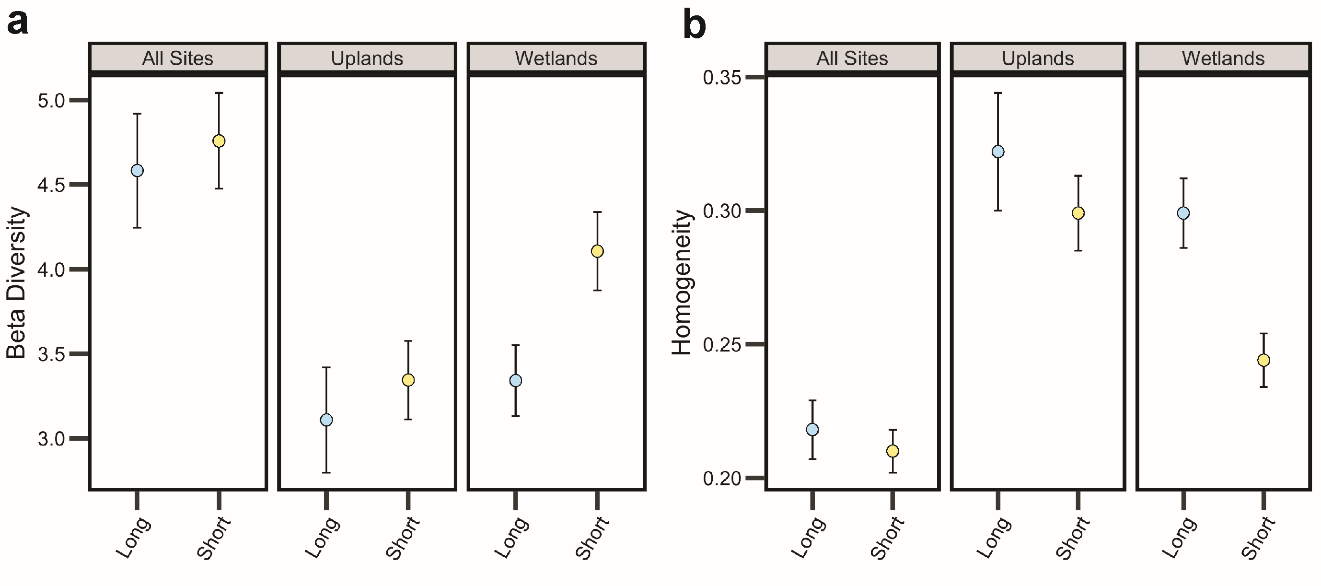


Figure S2. Hill’s numbers (calculated using Shannon measures) and 95% confidence intervals for understory vegetation community data, including (a) beta diversity, and (b) homogeneity.

**
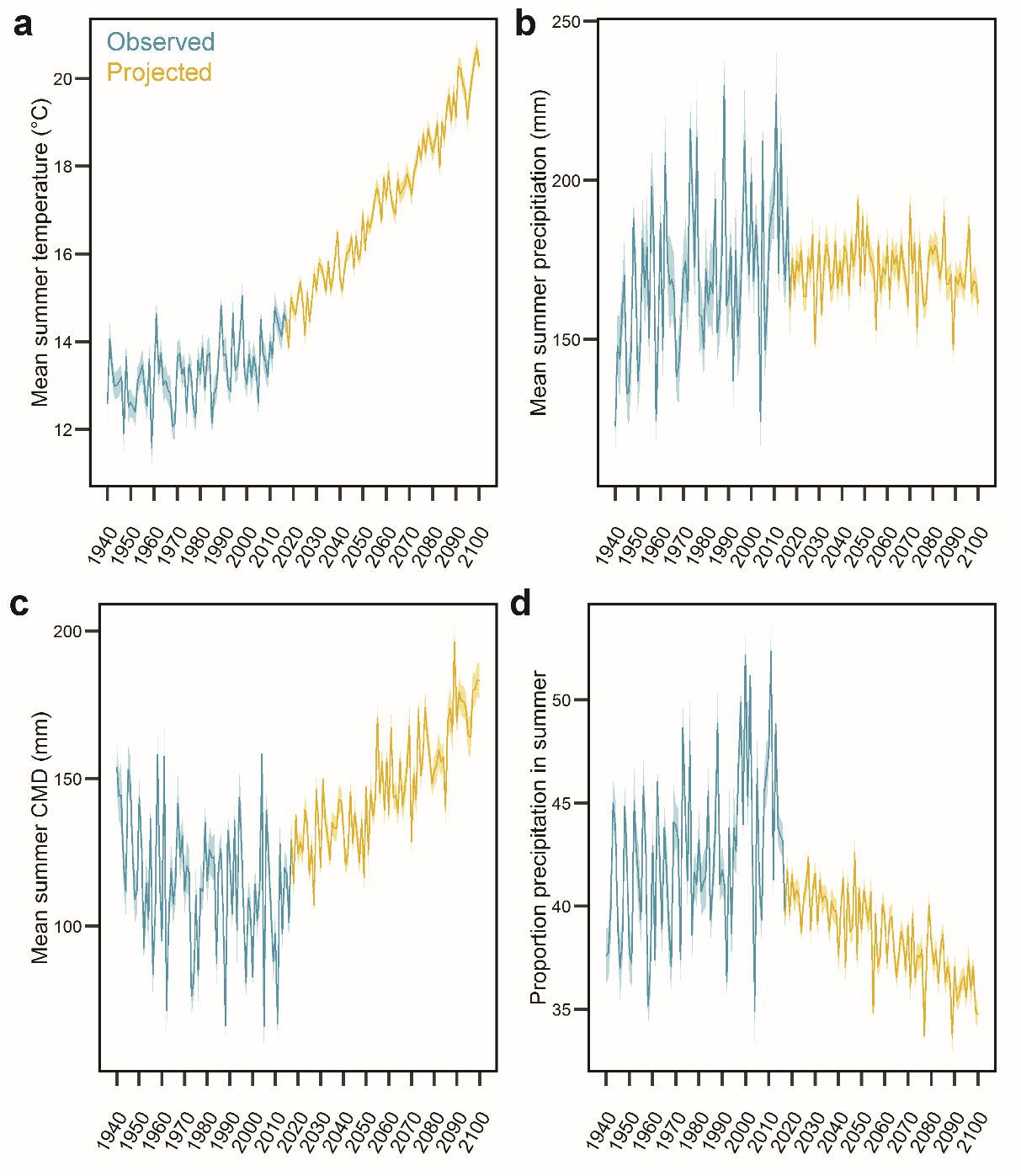
**

Figure S3. Observed historical summer and projected future climate (RCP 8.5) across the taiga plains and the western portion of the boreal plains ecoregion. Historical climate variables were downscaled from PRISM using ClimateWNA (4) and a digital elevation model (5). Projected climate conditions are an average of an ensemble of projections from 5 global circulation models (GCMs): ACCESS1.0, CanESM2, CNRM-CM5, CCSM4, and CSIRO Mk 3.6. Shaded areas behind darker lines represent the 95% confidence interval for the mean. Climate variables displayed are: (a) Mean summer temperature (ºC), (b) Mean summer precipitation (mm), (c) Mean summer climatic moisture deficit (CMD; mm), and (d) the mean proportion of total annual precipitation occurring in the summer months.


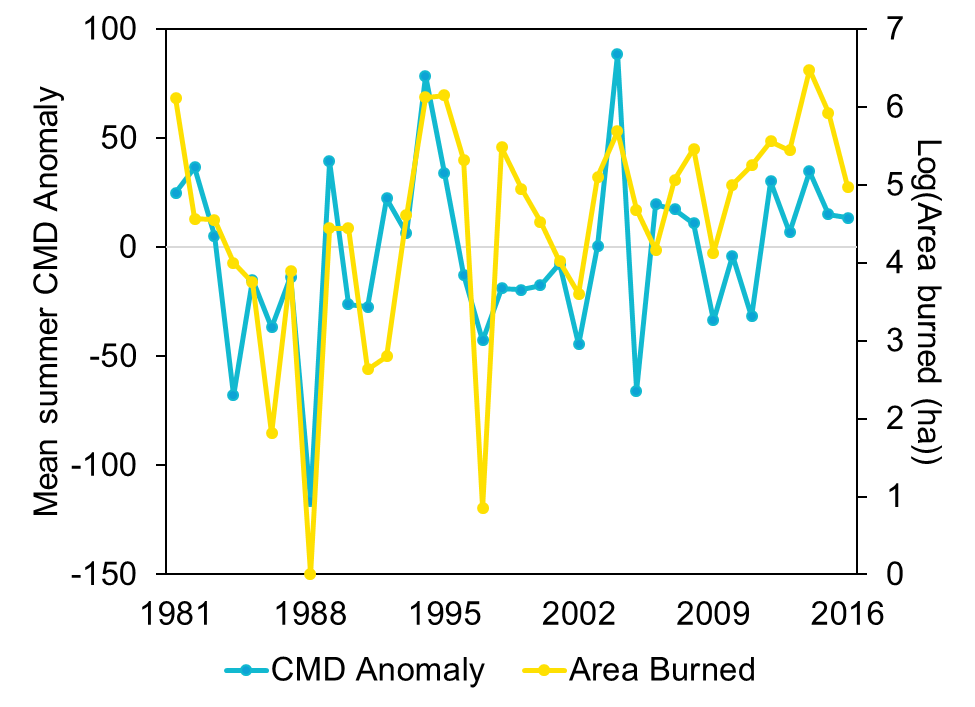


Figure S4. Temporal correlation (Spearman’s ρ = 0.6) between annual summer climatic moisture deficit (CMD) anomaly relative to a 30-year mean of summer CMD at sampled sites, and annual area burned (ha) within 150 km distance from field sites.­ The reference period for each year’s anomaly is the 30 years prior

Table S1. Ecological characteristics of sampled sites, and distribution of time since last fire (TSF) and fire-free interval (FFI).

| **Pair name** | **Hydrology** | **Ecosite** | **Prior Fire Year Long**  **(FFI)** | **Prior Fire Year Short (FFI)** | **Recent Fire Year** | **TSF at sample date (**$\bar{\boldsymbol{x}}$**=6.4)** |
| --- | --- | --- | --- | --- | --- | --- |
| 16-NT-11 | Upland | C1 Labrador Tea – Jack Pine/Black Spruce | 1942 (66) | 1998 (10) | 2008 | 8 |
| 16-NT-12 | Wetland | F3 Horsetail – White Spruce | 1973 (35) | 1998 (10) | 2008 | 8 |
| 16-NT-13 | Wetland | E2 Dogwoood – Jack Pine/White Spruce | 1947 (61) | 1998 (10) | 2008 | 8 |
| 16-NT-16 | Upland | C1 Labrador Tea – Jack Pine/Black Spruce | 1901 (107) | 1999 (9) | 2008 | 8 |
| 16-NT-17 | Upland | A1 Lichen- Jack Pine | 1900 (108) | 1999 (9) | 2008 | 8 |
| 16-NT-18 | Wetland | J1 Treed poor fen | 1903 (112) | 2011 (4) | 2015 | 1 |
| 16-NT-19 | Upland | A1 Lichen – Jack Pine | 1976 (39) | 1998 (17) | 2015 | 1 |
| 16-NT-20 | Wetland | J1 Treed poor fen | 1976 (39) | 1998 (17) | 2015 | 1 |
| 16-NT-21 | Upland | B1 Blueberry – Jack Pine/ Aspen | 1921 (74) | 1980 (15) | 1995 | 21 |
| 16-NT-22 | Wetland | K1 Treed rich fen | 1965 (30) | 1980 (15) | 1995 | 21 |
| 16-NT-23 | Wetland | K1 Treed rich fen | 1940 (55) | 1980 (15) | 1995 | 21 |
| 16-NT-24 | Wetland | I1 Treed bog | 1944 (51) | 1980 (15) | 1995 | 21 |
| 16-WB-01 | Upland | C1 Labrador Tea – Jack Pine/Black Spruce | 1980 (35) | 2011 (4) | 2015 | 1 |
| 16-WB-02 | Upland | A1 Lichen – Jack Pine | 1916 (97) | 2005 (8) | 2013 | 3 |
| 16-WB-03 | Upland | A1 Lichen – Jack Pine | 1912 (102) | 2004 (10) | 2014 | 2 |
| 16-WB-04 | Upland | A1 Lichen – Jack Pine | 1919 (95) | 2004 (10) | 2014 | 2 |
| 16-WB-05 | Upland | D2 Low-Bush Cranberry – Aspen/White Spruce | 1934^[[1]](#footnote-1)^ (80) | 2005 (9) | 2014 | 2 |
| 16-WB-06 | Upland | D2 Low-Bush Cranberry – Aspen/White Spruce | 1983 (32) | 2005 (9) | 2014, 2015 | 1,2 |
| 16-WB-07 | Upland | C1 Labrador Tea – Jack Pine/Black Spruce | 1961 (54) | 2004 (11) | 2015 | 1 |
| 16-WB-08 | Wetland | J1 Treed poor fen | 1925 (90) | 2004 (11) | 2015 | 1 |
| 16-WB-09 | Upland | A1 Lichen – Jack Pine | 1969 (35) | 1998 (6) | 2004 | 12 |
| 16-WB-10A | Upland | B1 Blueberry – Jack Pine/ Aspen | 1977 (37) | 2004 (10) | 2014 | 2 |
| 16-WB-10B | Upland | B1 Blueberry – Jack Pine/ Aspen | 1977 (37) | 2004 (10) | 2014 | 2 |
| 16-WB-14 | Upland | B1 Blueberry – Jack Pine/ Aspen | 1950 (64) | 1998 (16) | 2014 | 2 |
| 16-WB-15 | Upland | B1 Blueberry – Jack Pine/ Aspen | 1950 (64) | 1998 (16) | 2014 | 2 |

Table S2. Pairwise correlation coefficients (Spearman’s ρ) between short- and long-interval pairs.

| **Variable** | **ρ** |
| --- | --- |
| Pre-fire proportion conifer stems | 0.6 |
| Pre-fire basal area (BA) | 0.57 |
| Moisture regime (subxeric to subhydric) | 0.83 |
| Summer climatic moisture deficit (CMD) (1961 – 1990 Normals) | 1 |
| Post-fire moisture stress (CMD anomaly) | 0.97 |

Table S3. Paired statistical tests of differences in environmental variables between paired short FFI and long FFI sites. We conducted tests with all pairs for which data exists (e.g., some sites had no residual organic soil and thus are excluded from testing of effects on organic soil), and with subgroups of upland and wetland sites. Tests for which we report the *t* statistic are paired student’s *t*-tests, and tests for which we report the *V* statistic are nonparametric Wilcoxon signed-rank tests. Significant results (*p* ≤ 0.05) are bolded, and *p*-values with a false-discovery rate correction for multiple comparisons (Benjamini & Hochberg. 1995) are reported in *p_adj_*. Grey cells indicate that only three sites make up this dataset, therefore we did not conduct any statistical tests.

| **Hypothesis and Response** | **All data** | | |  | **Uplands** | | |  | **Wetlands** | | |  |
| --- | --- | --- | --- | --- | --- | --- | --- | --- | --- | --- | --- | --- |
|  | **N pairs** | **Test Statistic** | ***P*** | ***P_adj_*** | **N pairs** | **Test Statistic** | ***P*** | ***P_adj_*** | **N pairs** | **Test Statistic** | ***P*** | ***P_adj_*** |
| Canopy density (%) is lower in short FFI sites. | 25 | *V* = 288 | **<0.001** | **0.006** | 17 | *V* = 145 | **<0.001** | **0.009** | 8 | *V* = 28 | **0.01** | **0.015** |
| Biomass of rotten coarse woody debris (kg/m^2^) is lower in short FFI sites. | 25 | *V* = 249 | **0.002** | **0.007** | 17 | *V* = 126 | **0.002** | **0.009** | 8 | *V* = 20 | 0.42 | 0.525 |
| Mean residual organic soil depth (cm) is shallower in short FFI sites. | 25 | *t* = 2.76 | **0.005** | **0.012** | 17 | *t* = 0.34 | **0.02** | **0.04** | 8 | *t* = 1.78 | 0.06 | 0.15 |
| More exposed mineral soil (% cover) is present post-fire in short FFI sites. | 25 | *V* = 22 | **0.003** | **0.009** | 17 | *V* = 20 | **0.003** | **0.009** | 8 | *V* = 0 | 0.5 | 0.576 |
| Less surface organic matter (% cover) remains post-fire in short FFI sites. | 25 | *V* = 230 | **0.04** | 0.052 | 17 | *V* = 20 | **0.003** | **0.009** | 8 | *V* = 15 | 0.37 | 0.505 |
| Post-fire mineral soil pH is higher in short FFI sites. | 20 | *V* = 58 | **0.04** | 0.052 | 17 | *V* = 46 | 0.08 | 0.105 |  |  |  |  |
| Post-fire mineral soil total nitrogen (% N) is lower in short FFI sites | 20 | *V* = 162 | **0.02** | **0.034** | 17 | *V* = 105 | 0.09 | 0.109 |  |  |  |  |
| Residual organic soil total carbon (log(% C)) is lower in short FFI sites. | 21 | *t* = 1.73 | **0.05** | 0.061 | 13 | *t* = 1.51 | 0.08 | 0.105 | 8 | *t* = 0.81 | 0.22 | 0.413 |

Table S4. Paired statistical tests of differences in post-fire seedling and propagule cohorts between paired short FFI and long FFI sites. We conducted tests with all pairs for which data exists, and with subsets of upland and wetland sites. Tests for which we report the *t* statistic are paired student’s *t*-tests, and tests for which we report the *V* statistic are nonparametric Wilcoxon signed-rank tests. Significant results (*p* ≤ 0.05) are bolded, and *p*-values with a false-discovery rate correction for multiple comparisons (Benjamini & Hochberg, 1995) are reported in *p_adj_*.

| **Hypothesis and Response** | **All data** | | |  | **Uplands** | | |  | **Wetlands** | | |  |
| --- | --- | --- | --- | --- | --- | --- | --- | --- | --- | --- | --- | --- |
|  | **N pairs** | **Test Statistic** | ***P*** | ***P_adj_*** | **N pairs** | **Test Statistic** | ***P*** | ***P_adj_*** | **N pairs** | **Test Statistic** | ***P*** | ***P_adj_*** |
| Post-fire seedling and propagule density log(stems/m^2^) is lower in short FFI sites. | 24 | *V* = 233 | **0.002** | **0.007** | 17 | *V* = 123 | **0.01** | **0.024** | 7 | *V* = 20 | **0.03** | 0.112 |
| Post-fire conifer seedling density log(stems/m^2^) is lower in short FFI sites. | 24 | *V* = 255 | **<0.001** | **0.006** | 17 | *V* = 138 | **0.001** | **0.008** | 7 | *V* = 21 | **0.02** | 0.100 |
| Post-fire broadleaf seedling and propagule density log(stems/m^2^) is lower in short FFI sites. | 24 | *t* = -0.16 | 0.44 | 0.467 | 17 | *t* = -0.22 | 0.42 | 0.430 | 7 | *t* = 0.19 | 0.6 | 0.600 |
| Post-fire broadleaf seedling and propagule density log(stems/m^2^) is higher in short FFI sites. | 24 | *t* = -0.16 | 0.56 | 0.560 | 17 | *t* = 0.19 | 0.43 | 0.430 | 7 | *t* = -0.22 | 0.6 | 0.600 |
| The proportion of conifer stems in the post-fire cohort is lower in short FFI sites. | 24 | *V* = 214 | **<0.001** | **0.006** | 17 | *V* = 125 | **0.002** | **0.008** | 7 | *V* = 14 | **0.05** | 0.15 |

Table S5. Percentage variable contribution of explanatory variables to linear models.

| **Model** | **Variable** | **AIC** | **ΔAIC** | **% Contribution** |
| --- | --- | --- | --- | --- |
| Total post-fire stem density | Full model | 431.21 |  |  |
|  | Fire-free interval (FFI) | 450.07 | 18.86 | 82.86 |
|  | Site moisture (MOIST) | 435.07 | 3.86 | 16.96 |
|  | Moisture stress (MS) | 431.25 | 0.04 | 0.20 |
|  | Total |  | 22.76 |  |
| Conifer post-fire seedling density | Full model | 369.97 |  |  |
|  | Fire-free interval (FFI) | 384.11 | 14.14 | 63.021 |
|  | Pre-fire percent conifer (%CON) | 375.47 | 5.5 | 24.51 |
|  | Residual organic matter (RO) | 371.43 | 1.46 | 6.51 |
|  | Time since last fire (TSF) | 371.07 | 1.1 | 4.91 |
|  | Moisture stress (MS) | 370.21 | 0.24 | 1.07 |
|  | Total |  | 22.44 |  |
| Broadleaf post-fire stem density | Full model | 418.82 |  |  |
|  | Percent exposed mineral soil and rock (%MIN) | 589.15 | 170.33 | 36.16 |
|  | Residual organic matter (RO) | 556.12 | 137.3 | 29.14 |
|  | Moisture stress (MS) | 483.62 | 65.62 | 13.93 |
|  | Time since last fire (TSF) | 480.49 | 61.67 | 13.09 |
|  | Pre-fire basal area of trees (BA) | 455.01 | 36.19 | 7.68 |
|  | Total |  | 471.11 |  |
| Proportion conifer stems | Full model | 54.225 |  |  |
|  | Fire-free interval (FFI) | 66.011 | 11.786 | 59.54 |
|  | Residual organic matter (RO) | 58.406 | 4.181 | 21.12 |
|  | Percent exposed mineral soil and rock (%MIN) | 57.204 | 2.979 | 18.04 |
|  | Moisture stress (MS) | 55.08 | 0.855 | 4.32 |
|  | Total |  | 19.801 |  |

Table S6. Percentage variable contribution of explanatory variables to linear models, with variance explained by interactions partitioned from main effects. Variables with negative ΔAIC values are retained to demonstrate that the variable is not significant on its own, but are not included in the total sum of ΔAIC**.**

| **Model** | **Variable** | **AIC** | **ΔAIC** | **% Contribution** |
| --- | --- | --- | --- | --- |
| Total post-fire stem density | Full model | 431.21 |  |  |
|  | Fire-free interval (FFI) | 445.65 | 14.44 | 71.03 |
|  | Site moisture (MOIST) | 435.07 | 3.86 | 18.99 |
|  | FFI × MS | 433.24 | 2.03 | 9.99 |
|  | Moisture stress (MS) | 429.25 | -1.96 | 0 |
|  | Total |  | 20.33 |  |
| Conifer post-fire seedling density | Full model | 369.97 |  |  |
|  | Fire-free interval (FFI) | 382.31 | 12.34 | 51.91 |
|  | Pre-fire percent conifer (%CON) | 375.47 | 5.5 | 23.14 |
|  | RO × TSF | 373.03 | 3.06 | 12.87 |
|  | Residual organic matter (RO) | 368.57 | 1.4 | 5.89 |
|  | Time since last fire (TSF) | 370.69 | 0.79 | 3.32 |
|  | Moisture stress (MS) | 370.56 | 0.59 | 2.48 |
|  | FFI × MS | 370.06 | 0.09 | 0.4 |
|  | Total |  | 23.77 |  |
| Broadleaf post-fire stem density | Full model | 418.82 |  |  |
|  | Percent exposed mineral soil and rock (%MIN) | 543.38 | 124.56 | 30.37 |
|  | Residual organic matter (RO) | 502.87 | 84.05 | 20.5 |
|  | Time since last fire (TSF) | 480.49 | 61.67 | 15.04 |
|  | RO × MS | 475.75 | 56.18 | 13.7 |
|  | MIN × BA | 447.42 | 28.6 | 6.97 |
|  | Moisture stress (MS) | 446.9 | 28.08 | 6.85 |
|  | Pre-fire basal area of trees (BA) | 445.78 | 26.96 | 6.57 |
|  | Total |  | 410.1 |  |
| Proportion conifer stems | Full model | 54.225 |  |  |
|  | Fire-free interval (FFI) | 66.011 | 11.786 | 53.82 |
|  | Residual organic matter (RO) | 58.406 | 4.181 | 19.09 |
|  | Percent exposed mineral soil and rock (%MIN) | 57.263 | 3.038 | 13.87 |
|  | %MIN × MS | 57.116 | 2.891 | 13.20 |
|  | Moisture stress (MS) | 53.117 | -1.108 | 0 |
|  | Total |  | 21.898 |  |

Table S7. Paired statistical tests of differences in post-fire understory vegetation communities between paired short FFI and long FFI sites. We conducted tests with all pairs for which data exists, and with subsets of upland and wetland sites. Tests for which we report the *V* statistic are nonparametric Wilcoxon signed-rank tests. Significant results (*p* ≤ 0.05) are bolded, and *p*-values with a false-discovery rate correction for multiple comparisons (Benjamini & Hochberg, 1995) are reported in *P_adj_*.

| **Hypothesis and Response** | **All data** | | |  | **Uplands** | | |  | **Wetlands** | | |  |
| --- | --- | --- | --- | --- | --- | --- | --- | --- | --- | --- | --- | --- |
|  | **N pairs** | **Test Statistic** | ***P*** | ***P_adj_*** | **N pairs** | **Test Statistic** | ***P*** | ***P_adj_*** | **N pairs** | **Test Statistic** | ***P*** | ***P_adj_*** |
| Tall shrub density log(shrubs/m^2^) is higher in short FFI sites. | 23 | *V* = 76 | 0.09 | 0.102 | 16 | *V* = 26 | **0.05** | 0.077 | 7 | *V* = 11 | 0.34 | 0.505 |
| Cover of herbaceous plants (%) is lower in short FFI sites. | 25 | *V* = 239 | **0.02** | **0.034** | 17 | *V* = 120 | **0.02** | **0.04** | 7 | *V* = 22 | 0.32 | 0.505 |
| Cover of forbs (%) is lower in short FFI sites. | 25 | *V* = 247 | **0.01** | **0.021** | 17 | *V* = 113 | **0.04** | 0.068 | 7 | *V* = 29 | 0.07 | 0.15 |
| The proportion of herbaceous vegetation cover contributed by graminoids is higher in short FFI sites. | 25 | *V* = 97 | **0.04** | 0.052 | 17 | *V* = 57 | 0.19 | 0.215 | 7 | *V* = 3 | **0.02** | 0.10 |

Table S8. Significant indicator species of short and long interval uplands and wetlands identified using multilevel pattern analysis, and their regeneration strategies and fire tolerance. Significance levels are indicated by asterisks after the species name (*p* = 0.05 *, *p* = 0.01 **). We identified plant species traits from the Fire Effects Information System (FEIS; https://www.feis-crs.org/feis/; 1).

| **Indicator Species** | **Reproduction** | **Post-fire regeneration strategy** | **Resprouting depth** | **Shade Tolerance** | **Leaf type** | **Fire Tolerance** |
| --- | --- | --- | --- | --- | --- | --- |
|  | **Long FFI uplands (*n*=17)** | | | | |  |
| *Linnaea* borealis L. **** | Stolons & less commonly seed | Surface rhizome & off-site seed | Surface rhizome | All light conditions | Evergreen shrub | Fire intolerant |
| *Dracocephalum parviflorum* Nutt. **** | Soil seedbanking | Ground residual colonizer | NA | Partial shade to full light | Deciduous forb | Fire-stimulated germinant |
| *Geranium bicknellii* Britton *** | Soil seedbanking | Ground residual colonizer | NA | Shade intolerant | Deciduous forb | Fire-stimulated germinant |
| *Arctostaphylos uva-ursi* (L.) Spreng. *** | Stolons & shallow seedbank | Ground residual colonizer, Initial off-site colonizer | Shallow root crown | Shade intolerant | Evergreen shrub | Tolerant of moderate intensity fire, depending on rooting depth |
|  | **Short FFI uplands (*n*=17)** | | | | |  |
| *Leymus innovatus* (Beal) Pilg. *** | Rhizomes | Rhizome in soil | Deep rhizome | Partial shade to shade intolerant | Deciduous graminoid | Fire tolerant |
|  | **Long FFI wetlands (*n*=8)** | | | | |  |
| *Salix sp. ** ^^[[2]](#footnote-2)^†^ | Adventitious bud, root crown | Vigorous resprouting, off-site seed | Stems, root crown, and shallow to deep rhizomes | Partial shade to full light | Deciduous shrub | Tolerant of moderate to high intensity fire and repeated fire |
| *Rhododendron groenlandiucm* (Oeder) Kron & Judd *** | Root crown, rhizome, wind-dispersed seed | Bud & root crown, on-site or off-site seed | Stems, root crown, and shallow to deep rhizomes | Partial shade to full light | Evergreen shrub | Tolerant of low to moderate intensity fire, depending on rooting depth |
| *Equisetum arvense* L. *** | Rhizomes | Rhizome in soil, ground residual colonizer, initial off-site colonizer | Deep rhizome | All light conditions | Deciduous forb | Fire tolerant |
| *Chamaedaphne calyculata* (L.) Moench*** | Rhizomes, seed | Rhizome at surface or in soil, off-site seed | Deep rhizome | Shade intolerant | Evergreen shrub | Fire tolerant |
| *Vaccinium uliginosum* L. *** | Seed, layering, rhizomes | Survivor species, Rhizome or root crown, off-site seed | Shallow to deep rhizome in organic layer | Partial shade to full light | Deciduous shrub | Tolerant of moderate to high-intensity fire that does not completely consume organic layer |
| **Short FFI wetlands (*n*=8)** | | | | | | |
| *Betula pumila* L.**** | Layering, root crown, rhizomes, seed | Adventitious buds, rhizome or root crown, off-site colonizer | Deep root and rhizome | Shade intolerant | Deciduous shrub | Tolerant of low to moderate intensity fire |
| *Salix sp ** | Adventitious bud, root crown | Vigorous resprouting, off-site seed | Stems, root crown, and shallow to deep rhizomes | Partial shade to full light | Deciduous shrub | Tolerant of moderate to high intensity fire and repeated fire |
| *Epilobium palustre* L. *** ^^[[3]](#footnote-3)^‡^ (2, 3) | Stolons & slender rhizomes, seed | Rhizome, seed bank, off-site seed | Unknown | Partial shade to full light | Deciduous forb | Unknown |

**References**

1. USDA Forest Service Rocky Mountain Research Station Fire Scienes Laboratory (2018) Fire Effects Information System (FEIS). Available at: https://www.feis-crs.org/feis/ [Accessed December 4, 2018].

2. Douglas G, Meidinger D, Pojar J eds. (1999). *Illustrated Flora of British Columbia. Volume 3: Dicotyledons (Diapensiaceae Through Onagraceae)* (B.C. Minstry of Environment, Lands & Parks and B.C. Ministry of Forests, Victoria, BC).

3. van der Valk AG, Verhoeven JTA (1988) Potential role of seed banks and understory species in restoring quaking fens from floating forests. *Vegetatio* 76(1–2):3–13.

4. Wang T, Hamann A, Spittlehouse D, Carroll C (2016) Locally downscaled and spatially customizable climate data for historical and future periods for North America. *PLoS One* 11(6):1–17.

5. Natural Resources Canada (2016) Canadian Digital Elevation Model. Available at: http://ftp.geogratis.gc.ca/pub/nrcan_rncan/elevation/cdem_mnec/ [Accessed November 14, 2018].

1. Establishment date of non-serotinous tree (*Picea glauca*), not necessarily from fire origin. [↑](#footnote-ref-1)
2. † *Salix sp.* Characteristics were generalized from *Salix arbusculoides*, *S. fuscescens*, *S. glauca*, *S. lucida*, *S. myrtillifolia*, *S. planifolia*, and *S. scouleriana*. [↑](#footnote-ref-2)
3. ‡ Species not found in FEIS. [↑](#footnote-ref-3)
